# Dental and periodontal status of 12-year-old Dai school children in Yunnan Province, China: a cross-sectional study

**DOI:** 10.1186/s12903-015-0106-7

**Published:** 2015-10-08

**Authors:** Shinan Zhang, Biao Xu, Juan Liu, Edward CM Lo, Chun-Hung Chu

**Affiliations:** Faculty of Stomatology, Kunming Medical University, Yunnan, China; Faculty of Dentistry, The University of Hong Kong, Hong Kong, China

**Keywords:** Caries, Children, Ethnic, Minority, China

## Abstract

**Background:**

The Dai people are one of the ethnic minorities in China and have a population of 1,260,000. They have the same origin as one of the main ethnic groups in Laos and Thailand. The study aims to describe the dental caries and gingival status of 12-year-old Dai children in China and to study the factors affecting their oral-health status.

**Methods:**

This cross-sectional oral-health survey was conducted from 2011–2012 with ethics approval. A sample of 12-year-old Dai children living in Yunnan, China, was selected using a multistage and cluster sampling method. One trained examiner performed the clinical examination. Caries experience was measured using DMFT index, and gingival status was assessed with CPI index. A self-completed questionnaire was sent to the children, and they were asked about their backgrounds and oral-health-related behaviors and oral-health knowledge. Binary logistic regression analysis was performed to investigate the factors that affected the caries status.

**Results:**

A total of 875 children were invited, and 823 (94 %) joined the survey. The prevalence of caries experience among the participants was 40 %. The mean DMFT and DT scores were 0.9 and 0.8, respectively. Most children (93 %) had gingivitis, and many (46 %) had calculus. Girls and those who had visited a dentist during the previous year had a higher prevalence of caries.

**Conclusion:**

Dental caries were prevalent among 12-year-old Dai children in China. The periodontal condition of most of the children was poor. Caries were associated with gender and dental attendance.

**Electronic supplementary material:**

The online version of this article (doi:10.1186/s12903-015-0106-7) contains supplementary material, which is available to authorized users.

## Background

Data from the latest national oral-health survey conducted in 2005 showed that the prevalence of caries among 12 year olds in China was 29 % [1]. The caries experience, as measured by mean DMFT score (*D* stands for decayed tooth, *M* denotes a missing tooth due to decay, and *F* represents a filled tooth). was 0.5, which is the lowest score reported among Western Pacific countries [2, 3]. However, there are still profound disparities within China. Only 20 % of all medical resources are located in rural areas, which is where the majority of ethnic minorities live.

China has 56 ethnic groups. The Han constitute approximately 92 % of the total population. Although the other 55 ethnic groups account for only 8 % of the total population of China, they number approximately 114 million [4]. They are widely scattered across the country, but the majority live in inland or border districts in the less-developed western region. These ethnic minorities are culturally and linguistically diverse. Studies have suggested that people from specific ethnic minorities often have poor oral health, and the impact of disparities associated with ethnic origin has been increasing [5, 6].

Twenty ethnic minorities in China have populations of more than 1 million people. The Dai people constitute one of these minorities, and their population is about 1,260,000 [7]. Most of the Dai people in China (97 %) live in the southwestern part of the subtropical valley areas of the Yunnan province, which is situated in the far southwestern part of mainland China [8].

The history of the Dai people in China can be traced back to 109 B.C., when they established their own country [8]. They have their own language, which belongs to the Chinese–Tibetan language family and is written in an alphabetic script. They also practice traditional Dai medicine, which is one of the four major types of Chinese medicine. Most Dai are Buddhists, which relates closely to their economic development and daily life. Most (91 %) Dai people in Yunnan live in rural areas, and about 81 % are engaged in farming. In 2010, the gross domestic product (GDP) per capita of the Dai autonomous prefectures was approximately U.S. $2,200, which is 84 % and 46 % of the provincial and national GDP, respectively [7, 9].

The World Health Organization (WHO) has selected 12 years as the indicator age group for international benchmarking of children’s oral health [10]. The authors of this paper conducted epidemiological surveys and reported on 12-year-old children in various regions and countries in Southeast Asia. These surveys aimed to help map out the caries prevalence and underlying factors of a range of ethnic populations, including southern Chinese children [11], Cambodian children [12], Filipino children [13] and Burmese children [14]. High caries rate and severe caries experience were reported among Dai preschool children [15]. However, epidemiological data on the caries status of Dai school children are scarce [16]. One study, using a small sample size, surveyed the oral health of Dai schoolchildren more than 20 years ago [16]. In order to better understand the dental health of Dai children in Yunnan, a new survey on a representative sample was warranted. The study described the dental caries and gingival status of 12-year-old Dai children in Yunnan, China and studied the factors affecting their oral health.

## Method

### Sample size and selection of children

This study was a cross-sectional survey carried out from 2011–2012 with ethics approval from the Institutional Review Board of the University of Hong Kong (IRB UW-11-377). As there were no recent data in the literature, the sample size was estimated based on the latest national oral-health survey conducted in 2005, when the prevalence of dental caries of 12-year-olds in western China was 25 %. We set the width of the 95 % confidence interval at 6 %; thus, the sample size required in this survey would be 800 (*n* = 4 × 1.96^2^ × p × [1-p]/L^2^; [*n*: no. of children, *p*: prevalence, and *L*: width of 95 % confidence interval]). We estimated that the response rate was 95 %; therefore, at least 842 children had to be recruited.

With the help of the Bureau of Public Health and the Bureau of Education of the local government, a sample of 12-year-old Dai children was selected using a multistage cluster-sampling method. In the first stage, 2 of the 16 Yunnan districts were chosen: a southern district (Xishuangbanna) and a western district (Lincang) in the Yunnan Province where the majority (more than 50 %) of the Dai people lived. In the second stage, 2 of the 3 counties in Xishuangbanna (Jinghong and Menghai) and 3 of the 8 counties in Lincang (Yun, Gengma and Shuangjiang), where the majority of Dai people resided, were selected. All the primary schools in the selected 5 counties were included in this survey. All 12-year-old Dai children in the schools were invited to participate in this study. Children with parental written informed consent, of Dai ethnic origin, and in good general health were included. Children who had major systemic diseases or who were on long-term medication, such as for epilepsy and systemic *lupus erythematosus*, were excluded from the study. Children who were absent from school on the day of examination were not included in this study.

### Questionnaire survey

Before conducting the survey, we paid a visit to each primary school to deliver the questionnaire and discuss its protocol. Invitation letters and consent forms were sent to the children’s parents, and their written informed consent was obtained before the study. The questionnaire used in this study was the same as the one used in our previous epidemiological studies [15, 17] (Additional file 1). The students answered the questionnaire by themselves at school under the supervision of a research assistant. The questionnaire included information on background (age and gender), oral-health-related behaviors (snacking habits, tooth-brushing practice, and dental visits), and oral-health knowledge of the surveyed students. Oral-health knowledge was measured using four questions on the causes and prevention of dental caries and gum diseases. For each question, up to 3 answers were accepted. A dental knowledge score was constructed by adding the total number of the correct responses. The score was in a ratio scale from 0 to 12, and a higher score indicated better dental knowledge.

### Clinical examination

All of the children underwent a clinical examination, which was conducted by a trained public-health dentist in the primary school using a ball-ended Community Periodontal Index (CPI) probe and a disposable dental mirror attached to an intra-oral light-emitting diode (LED) light. Dental caries status was assessed based on the methods and criteria recommended by the WHO [10]. The DMFT index was used to record the caries experience of permanent dentition [10]. Caries were detected mainly visually and recorded if a lesion had an unmistakable cavity, a shadow of discolored dentine visible through intact enamel, or a detectable softened floor or wall [10]. Signs of early caries, such as white or brown spot lesions and rough surfaces or fissures that were sticky to probing but without a detectable softened floor or wall, were not diagnosed as dental caries. The PA index, which is modified from the PUFA index (*P* denotes visible pulpal involvement; *U* represents ulceration caused by dislocated tooth fragments; *F* stands for fistula; *A* means abscess) [18], was used to record the presence of conditions resulting from severely untreated, decayed teeth. The PA index scores the presence of a pulp involvement, an abscess, or a fistula. *P* denotes an untreated, carious tooth with visible pulp involvement and was recorded when the pulp chamber was visible or when only the roots or root fragments remained. *A* denotes an untreated, carious tooth with visible apical infection that can take the form of a fistula or an abscess and was scored in the case of swelling containing pus or a released sinus tract related to a tooth with pulp involvement. Gingival status was measured using the CPI [10]. The scores are 0 for healthy, 1 for gingival bleeding, and 2 for calculus; the worst condition was recorded. The index teeth were the first four permanent molars (16, 26, 36, and 46), the upper right central incisor (11), and the lower left central incisor (31). Six sites for each tooth (mesio-buccal, mid-buccal, disto-buccal, mesio-lingual, mid-lingual, and disto-lingual) were examined. Calibration was conducted before the survey, and duplicated examination was carried out on the same day at a different time. As recommended by the WHO’s basic oral-health survey method [10], duplicate examinations were performed on 10 % of the children to evaluate the intra-examiner agreement on assessment during the course of a series of assessments. Those children were re-examined at least one hour after the first examination so that the examiner was unlikely to remember the oral-health status of the child.

### Data entry and analysis

Data processing and analysis were performed with IBM Statistical Product and Service Solutions (SPSS) Statistics 20.0. The distribution of the dental caries was not normal. The Mann–Whitney U test was used to analyze the differences in the severity of the dental caries between boys and girls. The caries prevalence between groups was assessed with a chi-square test. Binary logistic regression was performed to assess the effects of the independent variables studied, including the child’s gender, his or her oral-health-related behaviors, and knowledge on the child’s dental caries prevalence. The dependent variable was the presence or absence of dental caries, and all of the independent variables were entered into the model. The statistical significance level for all tests was set at 5 %.

## Results

A total of 875 children from 38 primary schools were invited and 823 children (399 boys and 424 girls) joined the survey. Forty-three children were absent on the day of examination and 7 children declined to participate. Two children who had epilepsy were excluded from the study. The response rate was 94 %. The kappa values representing the intra-examiner reliability for scoring DMFT, PA, and CPI were 0.96, 0.96, and 0.90, respectively.

A total of 40 % of the children examined (*n* = 330) had caries experience. The mean DMFT score was 0.9 (SD score of 1.5) (Table 1). Only 74 teeth with decay were restored. A majority of decayed teeth (88 %) were left untreated, and the highest DT score found in a child was 10. Higher DMFT scores were found for the girls than for the boys (1.1 ± 0.7 vs. 0.8 ± 1.2; p < 0.001). Girls also had higher DT and FT scores than the boys. More than one-third (36 %) of the carious teeth had progressed to pulp infection (PA > 0). The mean PA score for those children with caries was 0.2, which translates to an SD score of 0.6. A total of 17 % of the girls and 13 % of the boys had decayed teeth that had progressed to odontogenic infection (PA > 0).

**Table 1:** Caries prevalence, caries experience, decayed teeth, missing teeth, filled teeth, severe untreated caries and visible apical infection according to gender (*n* = 823)

Gender	n (%)	% caries	%PA	Mean DMFT(SD)	Mean DT(SD)	Mean MT(SD)	Mean FT(SD)	Mean PA(SD)	Mean A(SD)
Boys	399 (49 %)	37 %	13 %	0.8 (1.2)	0.7 (1.1)	<0.1 (0.2)	0.1 (0.3)	0.2 (0.5)	<0.1 (0.1)
Girls	424 (51 %)	43 %	17 %	1.1 (1.7)	0.9 (1.4)	<0.1 (0.2)	0.2 (0.8)	0.2 (0.6)	<0.1 (0.1)
Total	823 (100 %)	40 %	15 %	0.9 (1.5)	0.8 (1.3)	<0.1 (0.2)	0.1 (0.6)	0.2 (0.6)	<0.1 (0.1)
*p*-value		0.047	0.205	0.002	0.010	0.322	0.016	0.232	0.940

Among the surveyed children, 96 % (*n* = 766) brushed their teeth at least once daily, and around half of the girls (48 %, *n* = 198) reported a brushing habit of twice or more per day, which was higher than that of the boys (29 %, *n* = 112; Table 2). Daily snacking habits were common, and no significant difference of the daily snacking habits was found between boys and girls (Table 2).

**Table 2:** Tooth-brushing habit, snacking habit and dental visit behaviour according to gender

Factor (No.)	All (%)	Boys (%)	Girls (%)	*p*-value
Brushing twice or more daily (*n* = 798)				<0.001
Yes	310 (39 %)	112 (29 %)	198 (48 %)	
No	488 (61 %)	275 (71 %)	213 (52 %)	
Eating snacks daily (*n* = 797)				0.711
Yes	475 (60 %)	235 (60 %)	240 (59 %)	
No	322 (40 %)	155 (40 %)	167 (41 %)	
Visiting dentist within last year (*n* = 802)				0.708
Yes	233 (29 %)	116 (30 %)	117 (29 %)	
No	569 (71 %)	275 (70 %)	294 (72 %)	

Table 3 shows the relationship between the dental caries prevalence of the 12-year-old Dai children and the variables studied. A higher prevalence of dental caries was found among the girls than among the boys (49 % vs. 40 %, *p* = 0.017). Children eating or drinking sugary snacks daily had a higher prevalence of dental caries than those who did not (44 % vs. 35 %, *p* = 0.015).

**Table 3:** Caries prevalence according to the variables studied in the 12-year-old Dai children

Independent variable	DMFT > 0 (n, %)	*p*-value
Gender		
Boy	160 (40 %)	0.017
Girl	206 (49 %)	
Location of residence		0.169
Town	181 (47 %)	
Village	185 (43 %)	
Brush teeth twice or more daily		0.524
Yes	129 (42 %)	
No	192 (39 %)	
Take sugary snacks daily		0.015
Yes	208 (44 %)	
No	113 (35 %)	
Visit a dentist within last year		0.413
Yes	99 (43 %)	
No	224 (39 %)	

The three most commonly perceived causes for dental caries were sugar and sweet food, poor oral hygiene, and bacteria/plaque (Table 4). Approximately 10 % of the children were unaware of dental caries. Eating and drinking less sugar, rinsing with water after eating, and proper tooth-brushing techniques were the three most common answers for how to prevent dental caries. However, only 18 % of the children knew that fluoridated toothpaste could help prevent dental caries. The mean dental-knowledge score of the 12-year-old Dai children was 7.8 ± 3.6. Higher oral-health-related knowledge was found among the girls than the boys (8.1 ± 3.3 vs. 7.5 ± 3.8, *p* = 0.007). The odds of girls having dental caries experience (DMFT > 0) were 1.371 times greater (95 % C.I: 1.034–1.819) than those for boys. Having dental caries experience was significantly related to the frequency of dental visits (OR: 1.410, 95 % C.I: 1.056–1.883; Table 5).

**Table 4:** Perceived causes and preventive methods of dental caries (multiple response allowed)

Perceived cause of dental caries	Number	Percentage
Sugar and sweet food	619	77 %
Poor oral hygiene	406	51 %
Bacteria, plaque	322	40 %
Acid	197	25 %
Traditional Chinese belief (e.g. hot air)	74	9 %
Don’t know	57	7 %
Other reasons	2	0.2 %
Perceived method of caries prevention	Number	Percentage
Take less sugar	546	68 %
Rinse with water after eating	539	67 %
Brush teeth properly	358	45 %
Visit a dentist	154	19 %
Use fluoride toothpaste	143	18 %
Don’t know	39	5 %
Other method	1	0.1 %

**Table 5:** Relationship between dental caries prevalence of 12-year-old Dai children and selected independent variables (final model of binary logistic regression)

Independent variables	Group	Odds ratio	95 % C.I.	*p*-value
Gender	Girls	1.371	1.034-1.819	0.028
	Boys^a^			
Visited a dentist within last year	Yes	1.410	1.056-1.883	0.020
	No^a^			
Intercept		0.555		<0.001

^a^Reference category

Only 7 % of the surveyed children had healthy gums (highest CPI = 0; Table 6). Most of them (93 %, *n* = 762) had unhealthy gums (highest CPI > 0), and the mean number of sextants with bleeding gums was 5.1 (SD score of 1.1). There was no statistically significant difference in the distribution of the highest CPI scores between the boys and girls.

**Table 6:** Periodontal status according to gender (*n* = 823)

	Total	Boys	Girls	*p*-value
No. (%) of children				
Highest CPI = 0	61 (7 %)	31 (8 %)	30 (7 %)	0.704
Highest CPI = 1	388 (47 %)	183 (46 %)	205 (48 %)	0.476
Highest CPI = 2	374 (46 %)	185 (46 %)	189 (45 %)	0.606
Mean (SD) number of sextants				
Healthy (CPI = 0)	0.1 (0.4)	0.2 (0.4)	0.1 (0.4)	0.196
Bleeding only (CPI = 1)	5.1 (1.1)	5.0 (1.2)	5.1 (1.0)	0.336
With calculus (CPI = 2)	0.8 (1.0)	0.8 (1.1)	0.7 (1.0)	0.572

## Discussion

Oral health is an important, integral part of general health [10]. Epidemiological studies are highly relevant to the assessment of disease patterns and needs, and such information may thereby aid the planning and evaluation of healthcare programs. This study used multistage sampling, which generally is considered a more accurate sampling for the sample size [19]. In this multilevel cluster sampling, representative clusters are chosen, and every child within the chosen cluster is sampled. It is a convenient and effective method of finding the survey sample and is particularly suitable for this study, which requires a large sample size. It is noteworthy that children selected using the multistage cluster-sampling method may not be as representative as those selected through random sampling.

The intra-examiner consistency of recording dental caries was high because the survey was conducted by an experienced dentist with advanced training in epidemiology. In addition, the dentist performed calibration trials before the survey. The mean DMFT score of the Dai children in this study was higher than that of the 12-year-old children reported in the latest national oral-health survey of China [20]. This may be because most Dai people live in the subtropical zone where sugar is produced. The availability of sugar promotes its consumption in various forms such as in sweets and soft drinks. However, the caries experience of the children in this study was considered low when it was compared to the global standard of less than 3 DMFT by the year 2000 [21]. In this survey, the caries experience of the Dai 12-year-old children in Yunnan is lower than that of the children in Thailand [22]. This may be due to their different socioeconomic status and lifestyle. Nevertheless, the need for dental care was high because most of the caries found were untreated and the population of the Dai children was large. Organized oral-health promotion programs were uncommon in rural areas of China, especially in western areas where the economy was less developed. Oral-health promotion programs for the ethnic-minority-group children, such as school-based programs to promote a tooth-brushing habit with affordable fluoride toothpaste and use of various fluoride agents, should be developed. Besides preventive measures, the use of caries-arrest treatment, atraumatic restorative treatment, endodontic treatment, and even extraction should be provided for children who have caries.

Although dental caries are caused by plaque on the tooth surface, strong clinical evidence demonstrating that tooth brushing per se is effective in caries prevention is lacking [23]. This conclusion is in agreement with our study, as the Dai girls brushed their teeth more frequently than the boys (*p* < 0.001). However, higher DMFT scores were also found among the girls than among the boys. Higher DMFT scores among the girls compared to the boys were also found in the national oral-health survey and other studies conducted in other countries [20, 22]. This may be due to the earlier development and eruption of the permanent teeth in girls.

A higher caries rate was found in the children who visited a dentist. This is a common phenomenon for dental care focused on restorative treatment [12, 14]. Many parents would take a child to visit a dentist when a tooth was in pain, but they would not take their children to see a dentist for preventive care [15, 17]. There are a few unregistered, so-called dentists who have received training through apprenticeship in rural areas [24]. They provide dental care consisting mainly of the relief of pain and tooth extraction. The government should encourage more registered dentists to work in the rural areas or implement compulsory dental services that are similar to those in other Southeast Asian countries such as Malaysia and Singapore. Furthermore, it is essential to promote early and regular preventive dental visits among children through a primary healthcare approach, which uses appropriate technology, focuses on prevention, involves community efforts, and uses a multi-sectorial approach [25].

The CPI was assessed using indexed teeth based on the WHO’s recommendation in 1997. It is noteworthy that after this survey was done, the WHO recommended examining all teeth to assess the CPI [26]. The periodontal health status of the 12-year-old children in this survey was unsatisfactory which is not uncommon for Chinese school children [27]. Most children had gum bleeding upon probing, and only 7 % of the 12-year-old children in this survey had healthy gums. Most of the six sextants had bleeding gums, which indicates that they had generalized gingivitis. Furthermore, nearly half of them (45 %) had calculus on their teeth. This percentage is higher than that of the 12-year-old children in west China [20]. This could be related to their poor knowledge on periodontal disease. It should be noted that more than 90 % of the surveyed Dai school children reported that they brushed their teeth at least once a day [28]. These children did not acquire a proper tooth-brushing technique. The other reason could be that they gave a socially acceptable answer. This is not uncommon in the Chinese and Asian cultures [17].

## Conclusion

Dental caries were prevalent among 12-year-old Dai children in China. Many of the decayed teeth were left untreated, and some had odontogenic infections. Caries prevalence was associated with gender and dental attendance. Their periodontal condition was poor, and around half of them had calculus.

## Additional file

Additional file 1:
**Oral health survey of Dai children in Yunnan.** (DOCX 21.1 kb)
