# Supplementary material for: Dental and periodontal status of 12-year-old Dai school children in Yunnan Province, China: a cross-sectional study
Source: BMC Oral Health. 2015 Oct 8;15:117. doi: 10.1186/s12903-015-0106-7 (PMC4597448; doi:10.1186/s12903-015-0106-7)
Supplement: Additional file 1: — Oral health survey of Dai children in Yunnan. (DOCX 21.1 kb) [file 12903_2015_106_MOESM1_ESM.docx]

Case No:

**Oral health survey of Dai children in Yunnan**

1. Name：________________ Class：________________

2. Contact number：________________

3. Gender：□_1_ Male　□_2_ Female

4. Where does the child live?

□_1_ Town □_2_ Village

5. How frequent do you brush your teeth?

□_1_ Never/occasionally □_2_ Once a day □_3_ Twice or more a day

6. Do you take sweet snacks daily?

□_1_ Yes □_2_ No

7. Did you visit a dentist in the last 12 months?

□_1_ Yes □_2_ No

8. What do you think cause tooth decay?

(Up to three answers are accepted.)

| □_1_ Poor oral hygiene (No or poor toothbrushing)  □_2_ Sugar and sweet food  □_3_ Bacteria and plaque  □_4_ Acid  □_5_ Chinese explanation (e.g. hot air)  □_6_ Others, please specify ________________  □_7_ Don’t know |
| --- |

9. What do you think cause swollen or bleeding gums?

(Up to three answers are accepted.)

□_1_ Poor oral hygiene (No or poor toothbrushing)

□_2_ Inadequate nutrition, lack of vitamin C

□_3_ Bacteria and plaque

□_4_ Chinese explanation(e.g. hot air)

□_5_ Inadequate sleep

□_6_ Others, please specify ________________

□_7_ Don’t know

10. What do you think you can do to prevent tooth decay?

(Up to three answers are accepted.)

□_1_ Rinse mouth after eating

□_2_ Toothbrushing (more frequent or better)

□_3_ Use fluoridated toothpaste

□_4_ Take less sugar

□_5_ Visit a dentist

□_6_ Others, please specify ________________

□_7_ Don’t know

11. What do you think you can do to prevent swollen or bleeding gums?

(Up to three answers are accepted.)

□_1_ Toothbrushing (more frequent or better)

□_2_ Use medicated toothpaste

□_3_ Drink cooling tea or herbal medicine

□_4_ Visit a dentist

□_5_ Eat fruit/nourishing food

□_6_ Others, please specify ________________

□_7_ Don’t know

~ The End ~
